# Serum Leptin Concentrations in Turkish Parkinson's Disease Population

**DOI:** 10.1155/2014/576020

**Published:** 2014-04-28

**Authors:** Betul Ozdilek, Gulay Kenangil

**Affiliations:** Erenkoy Research and Training Hospital for Neurologic and Psychiatric Disorders, Department of Neurology, Sinan Ercan Caddesi No. 29, Erenkoy/Kadikoy, 34736 Istanbul, Turkey

## Abstract

*Objectives*. To investigate leptin levels and their relationship to body composition and demographic and clinical characteristics of Turkish patients with Parkinson's disease (PD). *Patients and Methods*. Forty eligible PD patients and 25 healthy controls were included in the study. Body composition measurements (height, weight, waist circumference (WC), and body mass index (BMI)) of the whole sample and clinical findings of PD patients were evaluated in the on-state. A single 5 mL fasting blood sample was obtained from each participant in the morning. Severity of PD was evaluated using the Hoehn and Yahr scale and the Unified Parkinson's Disease Rating Scale. *Results*. The mean age of the patients and controls was 60.8 ± 9.4 and 61.8 ± 5.8 years, while the mean BMI was 30.17 ± 5.10 and 28.03 ± 3.23 and the mean leptin levels were 6.8 ± 6.9 and 3.9 ± 3.8 ng/mL, respectively. Only age and gender were correlated with leptin levels. There was a significant difference (*P* < 0.001) in leptin levels between male (3.6 ± 3.1 ng/mL) and female (14.3 ± 7.7 ng/mL) PD patients. Among the male PD patients, older age and higher BMI and WC values were associated with higher mean leptin levels. There was not any significant relationship between leptin levels and clinical findings in PD patients. *Conclusion*. These results may suggest that leptin levels have no determinative role in the follow-up of PD patients with regard to the severity and clinical prognosis of PD.

## 1. Introduction

Parkinson's disease (PD) is an adult-onset, progressively evolving, neurodegenerative disease. It was initially known primarily as a motor disorder, with tremor, bradykinesia, rigidity, and postural instability as dominant features, caused by significant dopaminergic striatal denervation. Later work underscored the importance of nonmotor symptoms, including neuropsychiatric, autonomic, and gastrointestinal symptoms [1–6]. Both motor and nonmotor symptoms may influence the energy balance [7].

Leptin is a novel and promising molecule in research that may link body weight (BW), body mass index (BMI), and neurodegenerative diseases [8, 9]. Since the discovery of leptin in 1994, major advances have been made in understanding the neuroendocrine mechanisms regulating appetite, adiposity, obesity, metabolism, sympathetic tone, blood pressure, inflammation, and the hematopoietic and immune systems. Several studies have suggested that PD patients have lower BMI and serum leptin levels than controls [1, 10, 11]. However, differences between patients and controls were not statistically significant in these studies [12–14]. In fact, one uncontrolled study suggested that being overweight or obese may also be common in PD [15].

The present study was designed to examine the leptin profiles in Turkish PD patients and to ascertain any relationship of serum leptin levels with body composition variables (BW, BMI, and waist circumference (WC)) and clinical findings.

## 2. Materials and Methods

Forty eligible consecutive patients with PD (28 males, 12 females) attending the outpatient clinic and 25 healthy controls (14 males, 11 females) matched for sex, age, and body mass index (BMI) were enrolled into the study. Patients who were unable to ambulate, those with systemic illnesses such as diabetes, thyroid disease, and neoplasia, those with psychiatric diseases (psychosis and severe depression), those using any medication that may affect body weight and who had major dietary restrictions, and those who were demented (Mini-Mental State Examination, MMSE < 24) or institutionalized at the time of study entry were excluded. None of the patients presented with nausea or anorexia due to dopaminergic medication, and none changed their dietary habits throughout the whole study. No subject had undergone surgical treatment for PD.

The clinical type of PD was classified as tremor-predominant or akinesia/rigidity-predominant; dyskinesia status and postural instability were determined as present or absent. PD patients were evaluated in the on-state using the Hoehn and Yahr scale (HY) and the Unified Parkinson's Disease Rating Scale (UPDRS) parts I, II, III, and IV and total score [16]. All subjects were on various forms of dopaminergic treatment. L-dopa equivalent daily dosage (LEDD) was calculated and recorded [17].

At each clinical appointment, all patients underwent body composition measurements and blood sampling. The body composition measurements included body weight (BW; measured to the nearest 0.1 kg with the subject wearing a layer of clothing over underwear), height without shoes, waist circumference (WC), and BMI. BMI was calculated by dividing a direct weight measurement (in kilograms) by the squared average of at least two height measurements (in millimeters, converted to meters). These body composition parameters were also recorded for healthy controls.

A single 5 mL venous blood sample was collected from each fasting patient and control between 7:00 and 11:00 am. Serum was separated within 30 min and stored at −80°C until analysis for level of total leptin. Serum leptin concentrations were measured using the DIAsource ImmunoAssays human leptin ELISA kit (DIAsource, Nivelles, Belgium). This ELISA sandwiches human leptin between two monoclonal antibodies reacting against different epitopes on the leptin molecule.

The study protocol was approved by the Institutional Review Board. Informed consent was obtained from all subjects.

### 2.1. Statistical Analysis

All data were analyzed using the number cruncher statistical system (NCSS) 2007 and power analysis and sample size (PASS) 2008 statistical software packages (Utah, USA). Results are expressed as means ± standard deviation (SD), median, frequency, and rate. Unpaired *t*-tests and Spearman's correlation coefficients were calculated to assess intergroup differences and correlations, respectively. When comparisons were made between independent groups, the Mann-Whitney *U*-test was used. The Kruskal-Wallis test was used for comparisons of more than two groups (*r*, *P*). *P* values <0.05 were considered to indicate statistical significance. Multivariate linear regression analysis was performed to examine the association between leptin and those variables that were found to correlate significantly with leptin in the univariate analysis.

## 3. Results

The mean age of the PD patients and controls was 60.8 ± 9.4 and 61.8 ± 5.8 years, respectively. 70% of PD patients were males. In terms of body composition, the mean body weight (BW) of the patients was 81.05 ± 15.06 kg, BMI was 30.17 ± 5.10, and waist circumference (WC) was 106.58 ± 12.14 cm. The mean BMI of the controls was 28.03 ± 3.23. The mean serum leptin level for the PD patients and controls was 6.8 ± 6.9 ng/mL (range, 0.1–27.8 ng/mL) and 3.9 ± 3.8 (range, 0.1–15), respectively. The mean disease duration was 6.3 ± 4.5 years. The patients were equally distributed in HY stages 1, 2, and 3 (about 35% each). No patients had a severe disability, and none was bed ridden. Only five patients had a family history of PD. The mean LEDD was 714 ± 409 mg. Of the patients, 80% were on dopamine agonist treatment.  Table 1 shows the demographic profile and clinical characteristics, body composition, and serum leptin concentrations in male and female PD patients and controls.

**Figure 1:**
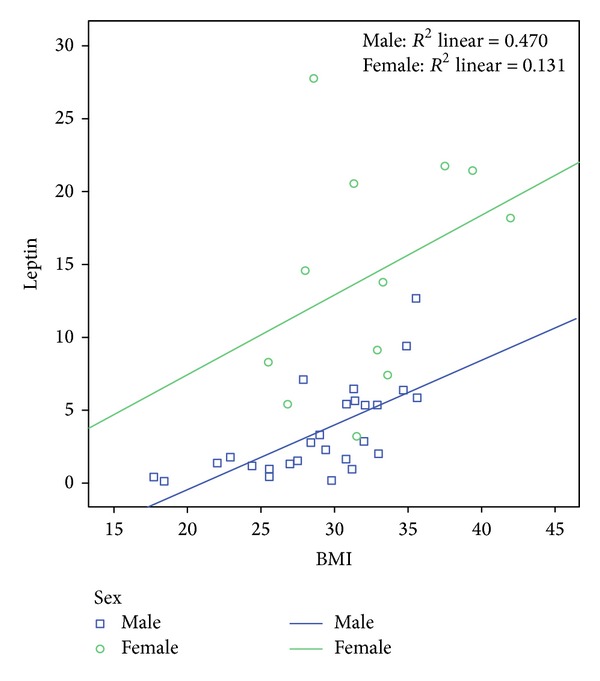
Correlation between leptin levels (ng/mL) with BMI (kg/m^2^) in male and female PD patients.

In PD patients, there was a significant difference (*P* < 0.001) in leptin concentrations between the male (3.6 ± 3.1 ng/mL) and female patients (14.3 ± 7.7 ng/mL). Serum leptin concentrations were positively correlated with BMI (Figure 1), BW, and WC in the study group as a whole and especially in male PD patients (Table 2). Body composition measurements and leptin levels of PD patients were significantly correlated (*P* < 0.05).

Age and gender of PD patients were analyzed for correlations with leptin levels, BMI, BW, and WC. Only leptin levels were associated with age and gender (but not with BW, BMI, or WC; Table 3). Among the male patients with PD, older age, higher BMI, and larger waist circumference were associated with higher mean leptin levels.

Clinical characteristics of PD, including disease duration, type of clinical onset, lateralization of symptoms, disease severity (expressed as HY stage), motor and nonmotor symptom severity (expressed as UPDRS scores), LEDD, and use of dopamine agonist were also analyzed for any correlation with leptin levels, BMI, or BW. The analysis was also repeated for female and male patients separately; no significant relationship between any of these variables and leptin levels was obtained (Table 3). Generally, higher leptin levels were associated with older age (*P* < 0.01), female gender (*P* < 0.01), greater BMI (*P* < 0.01), and use of dopamine agonist (*P* < 0.05).

## 4. Discussion

It is well established that the adipocyte-derived polypeptide hormone leptin is an important circulating satiety factor that regulates body weight and food intake via its actions on specific hypothalamic nuclei. However, there is growing evidence that leptin receptors are also widely expressed throughout the brain. Based on these findings, leptin concentrations may be considered a marker for the extent of body weight, obesity, and fat mass in humans. Excessive body fat accumulation and obesity are associated with increased levels of leptin. Thus, decreased leptin concentrations would be expected to be associated with decreased weight [18].

In addition to leptin levels in patients with PD, we also studied body weight (BW), BMI, and waist circumference (WC). Consistent with previous studies [1, 10, 11], we observed positive correlations between leptin levels and BMI, BW, and WC.

In agreement with previous studies [19, 20], the age of PD patients was correlated with leptin levels. Younger PD patients had lower mean leptin levels. Unlike some other studies, which found correlations between age and BW, BMI, and WC [13, 21], in our study, we could not find any correlation between age and body composition measurements, such as BW, BMI, and WC. There was not any correlation between leptin levels and demographic characteristics of PD patients such as gender, either.

Gender differences and their relationship with leptin levels and body composition have been examined in several studies. Leptin signaling may differ between the genders. In a recent study, it was reported that leptin levels are considerably lower in men than women, which is why it is important to report calculations of men and women separately. The reason for this finding is unclear; the larger amounts of body fat mass, predominantly subcutaneous fat, in women and the effects of sex steroid hormones that increase leptin production have been suggested [22, 23]. The present study confirms previous results regarding a gender difference in leptin levels between male and female patients with PD. We found significantly higher serum leptin levels in female than in male PD patients in this small sample. To our knowledge, this is the first such report in a PD population.

In previous studies [10, 11, 21, 22], leptin levels, BMI, and body weight were correlated with both motor and nonmotor PD symptoms as well as duration and clinical severity of the disease. Increased energy expenditure due to increased muscle tone, tremor, motor fluctuations, and dyskinesia has been proposed as a plausible mechanism to explain low BW. Also, nonmotor symptoms such as dysphagia, anorexia, and depression can affect BW [12, 19]. Thus, it would seem that weight loss is, at least in part, related to the clinical severity of PD. Treatment with levodopa, dopamine agonists, and other dopaminergic agents may play a role in the body weight of PD patients. Furthermore, a reduction in the total daily dose of L-dopa may have an additional role via probable lipolytic effects [13], as well as a central effect on the satiety/appetite hypothalamic centers [24, 25]. If so, L-dopa treatment could indirectly result in lower serum leptin levels. Ninety percent of our PD patients were on a dopamine agonist treatment. We think that the possible effects of dopamine agonists should also be investigated in larger groups further. Pooled data showed that BMI and BW decreased with longer disease duration and greater disease severity [21]. Determinants other than disease duration and severity were inconsistent, as reported in previous studies. Disease progression, characterized by weight loss and worsening of PD symptoms, has been proposed as an independent predictor of this weight loss [13, 21]. Surprisingly, disease duration, type of disease (tremor-dominant versus bradykinesia-dominant form), and UPDRS scores and HY stage, both markers of disease progression, were not significantly correlated with leptin levels, BMI, BW, or WC in our cohort. Only the use of dopamine agonist treatment was slightly associated with leptin levels.

Body weight, BMI, WC, and leptin levels differ in various populations due to their patterns of development. Hence, these changes may reflect differences in lifestyle, diet, physical activity, and other cultural and economic factors that are specific for people in this part of the world.

In conclusion, these results suggest that leptin levels were only affected by age and gender in PD patients. Disease duration, severity, and clinical status were not associated with leptin levels. Moreover, a slight relationship may exist between leptin levels and the use of dopamine agonists which needs to be investigated by further studies. From these results, we conclude that the measurement of serum leptin levels in PD patients has no determinative role with regard to the severity and clinical progression of PD. A longer follow-up of these patients is necessary to understand the leptinemia role during the worsening of neurological symptoms.

## Figures and Tables

**Table 1:** Sociodemographic and clinical characteristics of male and female PD patients and controls.

Characteristics	Male patients (*n* = 28)	Female patients (*n* = 12)	Male controls (*n* = 14)	Female controls (*n* = 11)
Age (years)	60.1 ± 9.0	62.4 ± 10.5	64.9 ± 5.3	57.9 ± 3.8
Education (years)	8.2 ± 3.9	7.1 ± 3.5	11.5 ± 4.3	10.6 ± 3.8
BMI (kg/m^2^)	29.1 ± 4.8	32.5 ± 5.0	27.3 ± 3.2	26.6 ± 3.3
BW (kg)	82.2 ± 15.3	78.2 ± 14.5	75.6 ± 8.8	67.8 ± 12.6
WC (cm)	105.1 ± 10.5	110.0 ± 15.1	95.7 ± 9.9	91.1 ± 13.5
Leptin (ng/mL)	3.6 ± 3.1	14.3 ± 7.7	1.7 ± 1.1	6.7 ± 4.3
Disease duration (years)	6.7 ± 4.9	5.3 ± 3.0	—	—
LEDD (mg/day)	820.6 ± 428.8	466.8 ± 218.0	—	—
UPDRS total scores	27.6 ± 17.1	21.9 ± 12.4	—	—

Results are expressed as means ± standard deviation.

LEDD: L-dopa equivalent daily dose; UPDRS: Unified Parkinson's Disease Rating Scale; BW: body weight; WC: waist circumference; BMI: body mass index.

**Table 2:** Correlation between leptin level and body composition measurements in male and female PD patients. BMI (kg/m^2^): body mass index; BW (kg): body weight; WC (cm): waist circumference.

Leptin level (ng/mL)				
		Female patients (*n* = 12)	Male patients (*n* = 28)	All subjects (*n* = 40)
BMI	*r *	0.329	0.731	0.624
	*P *	*0.297 *	0.001**	0.001**

BW	*r *	0.322	0.616	0.328
	*P *	*0.308 *	0.001**	0.039**

WC	*r *	0.266	0.557	0.471
	*P *	*0.404 *	0.002**	0.002**

Spearman correlation analysis.

**P < 0.01.

**Table 3:** Correlation of leptin levels, BMI, and WC with age, gender, and use of dopamine agonist treatment. BMI (kg/m^2^): body mass index; WC (cm): waist circumference.

		Leptin level	BMI	WC
Age	*r *	0.388	0.267	0.224
	*P*	^a^0.013*	^ b^ *0.095 *	^ b^ *0.165 *

Gender	*z *	−4.162	−1.565	−1.403
	*P*	^b^0.001**	^ b^ *0.118 *	^ b^ *0.160 *

Dopamine agonist use	*z *	−2.046	−0.457	−0.068
	*P*	^b^0.041*	^ b^ *0.648 *	^ b^ *0.946 *

^a^Spearman correlation analysis.

^b^Mann-Whitney *U*-test.

*P < 0.05.

**P < 0.01.
